# Supplementary material for: Similarities in Metabolic Flexibility and Hunger Hormone Ghrelin Exist between FTO Gene Variants in Response to an Acute Dietary Challenge
Source: Nutrients. 2019 Oct 18;11(10):2518. doi: 10.3390/nu11102518 (PMC6835809; doi:10.3390/nu11102518)
Supplement: Supplementary file 1 [file nutrients-11-02518-s001.zip › Sup Data/Manuscript - Peer Review Author Proofread Table S1.docx]

| **Outcome (between FTO genotypes)** |  | ***Covariant: BMI*** | |  | ***Covariant: Gender*** | |
| --- | --- | --- | --- | --- | --- | --- |
|  |  | ***P* value** | **R^2^** |  | ***P* value** | **R^2^** |
|  |  |  |  |  |  |  |
| Metabolic Flexibility |  | 0.329 | 0.043 |  | 0.357 | 0.040 |
| Energy Expenditure (kJ.kgLBM^-1^.min^-1^) |  |  |  |  |  |  |
| *Pre OGL* |  | 0.874 | 0.007 |  | 0.681 | 0.095 |
| *Post OGL (60 mins)* |  | 0.535 | 0.035 |  | 0.171 | 0.241 |
| Glucose Oxidation (g.kgLBM^-1^.min^-1^) |  |  |  |  |  |  |
| *Pre OGL* |  | 0.627 | 0.041 |  | 0.617 | 0.008 |
| *Post OGL (60 mins)* |  | 0.943 | 0.007 |  | 0.797 | 0.091 |
| Fat Oxidation (g.kgLBM^-1^.min^-1^) |  |  |  |  |  |  |
| *Pre OGL* |  | 0.493 | 0.056 |  | 0.361 | 0.054 |
| *Post OGL (60 mins)* |  | 0.820 | 0.005 |  | 0.771 | 0.008 |
| RER |  |  |  |  |  |  |
| *Pre OGL* |  | 0.323 | 0.036 |  | 0.372 | 0.073 |
| *Post OGL (60 mins)* |  | 0.728 | 0.012 |  | 0.827 | 0.020 |
| Plasma Glucose (mmol.L^-1^) |  |  |  |  |  |  |
| *Pre OGL* |  | 0.260 | 0.031 |  | 0.396 | 0.145 |
| *Post OGL (30 mins)* |  | 0.118 | 0.030 |  | 0.135 | 0.031 |
| *Post OGL (60 mins)* |  | 0.182 | 0.046 |  | 0.196 | 0.026 |
| *Post OGL (120 mins)* |  | 0.565 | 0.014 |  | 0.510 | 0.017 |
| Serum Ghrelin (Active) (pg.ml^-1^) |  |  |  |  |  |  |
| *Pre OGL* |  | 0.754 | 0.055 |  | 0.705 | 0.120 |
| *Post OGL (60 mins)* |  | 0.600 | 0.055 |  | 0.513 | 0.061 |
| *Post OGL (120 mins)* |  | 0.764 | 0.064 |  | 0.698 | 0.062 |
| Serum Insulin (µU.ml^-1^) |  |  |  |  |  |  |
| *Pre OGL* |  | 0.384 | 0.124 |  | 0.318 | 0.066 |
| *Post OGL (60 mins)* |  | 0.763 | 0.009 |  | 0.714 | 0.029 |
| *Post OGL (120 mins)* |  | 0.295 | 0.033 |  | 0.296 | 0.032 |
|  |  |  |  |  |  |  |
| HOMA-IR |  | 0.390 | 0.110 |  | 0.372 | 0.027 |
| ISI |  | 0.824 | 0.005 |  | 0.822 | 0.024 |
