# Supplementary material for: Similarities in Metabolic Flexibility and Hunger Hormone Ghrelin Exist between FTO Gene Variants in Response to an Acute Dietary Challenge
Source: Nutrients. 2019 Oct 18;11(10):2518. doi: 10.3390/nu11102518 (PMC6835809; doi:10.3390/nu11102518)
Supplement: Supplementary file 1 [file nutrients-11-02518-s001.zip › Sup Data/Manuscript - Peer Review Author Proofread Table S2.docx]

|  | **Partial eta-squared (ηp^2^)** | **Observed Power (ß)** | **Power (1-ß)** |
| --- | --- | --- | --- |
| RER | 0.020 | 0.310 | 0.690 |
| Energy Expenditure (kJ.kgLBM^-1^.min^-1^) | 0.006 | 0.112 | 0.888 |
| Glucose Oxidation (g.kgLBM^-1^.min^-1^) | 0.007 | 0.124 | 0.876 |
| Fat Oxidation (g.kgLBM^-1^.min^-1^) | 0.005 | 0.107 | 0.893 |
| Plasma Glucose (mmol.L^-1^) | 0.016 | 0.295 | 0.705 |
| Serum Insulin (µU.ml^-1^) | 0.015 | 0.205 | 0.795 |
| Serum Ghrelin (Active) (pg.ml^-1^) | 0.003 | 0.077 | 0.923 |
